# Antiosteoporosis effect and possible mechanisms of the ingredients of Radix Achyranthis Bidentatae in animal models of osteoporosis: systematic review and meta-analysis of in vivo studies

**DOI:** 10.1186/s13018-023-04031-w

**Published:** 2023-07-26

**Authors:** Yong Lian, Haoran Zhu, Xiaxia Guo, Yinuo Fan, Zhixing Xie, Jinfan Xu, Min Shao

**Affiliations:** 1 Department of Joint Diseases, The Third Affiliated Hospital of Guangzhou University of Chinese Medicine, NO. 261 Longxi Road, Liwan District, Guangzhou, Guangdong Province People’s Republic of China; 2 Department of Orthopedics, The Third Affiliated Hospital of Guangzhou University of Chinese Medicine, Guangzhou, Guangdong Province 510405 People’s Republic of China

## Abstract

**Background:**

The effect and mechanisms of the ingredients (IRAB) of Radix Achyranthis Bidentatae (RAB) on treating osteoporosis (OP) remains debated. We aimed to summary the evidence to evaluate the efficacy of IRAB for animal model OP and elucidate the potential mechanism of IRAB in the treatment of OP.

**Methods:**

In this review and meta-analysis, we searched PubMed, EMBASE, Web of Science, Cochrane Library, Chinese National Knowledge Infrastructure, Wanfang, Chinese Biomedical Literature Database, as well as Chinese VIP databases for targeting articles published from inception to March 2023 in English or Chinese. All randomized controlled animal trials that assessed the efficacy and safety of IRAB for OP were included. We excluded trials according to exclusion criteria. The CAMARADES 10-item quality checklist was utilized to test the risk of potential bias for each including study and modifications were performed accordingly. The primary outcome measures were bone mineral density of the femoral neck (F-BMD), serum calcium (Ca), serum phosphorus (P), serum alkaline phosphatase (ALP), bone gla protein (BGP), bone maximum stress (M-STRESS). The secondary outcome measure was the antiosteoporosis mechanisms of IRAB.

**Results:**

Data from nine articles were included in the systematic review and meta-analysis, which focused on 196 animals. Egger’s test revealed the presence of publication bias in various studies regarding the primary outcome. Administration of IRAB or RAB could significantly increases the F-BMD (SMD = 2.09; 95% CI = 1.29 to 2.89; *P* < 0.001, *I*^2^ = 76%), Ca (SMD = 0.86; 95% CI = 0.39to1.34; *P* = 0.07, *I*^2^ = 49%); P (SMD = 1.01; 95% CI = 0.45–4.57; *P* = 0.08, *I*^2^ = 50%), BGP (SMD = 2.13; 95% CI = 1.48 to 2.78; *I*^2^ = 46%, *P* = 0.10), while the ALP (SMD = − 0.85; 95% CI =  − 1.38 to − 0.31; *I*^2^ = 46%, *P* = 0.10) was remarkably decreased in OP model animals. Moreover, the bone biomechanical indicator M-STRESS (SMD = 2.39; 95% CI = 1.74–3.04; *I*^2^ = 32%, *P* = 0.21) was significantly improved.

**Conclusion:**

Collectively, the findings suggest that the RAB or IRAB could be an effective drug or an ingredient in diet for the clinical treatment of OP in future.

**Supplementary Information:**

The online version contains supplementary material available at 10.1186/s13018-023-04031-w.

## Introduction

Osteoporosis (OP) is a progressive degenerative bone diseases characterized by low bone mass and bone microarchitectural deterioration, with consequent increases in the fragility of bone and risk of fracture [1, 2]. Various risk factors may trigger this disease, such as age, diet, lack of exercise, low calcium and vitamin D levels, declining estrogen levels and some medication side-effects [3]. Many complications are associated with OP, including chronic pain, spinal deformities and fractures, which seriously influence the quality of life of elderly and health systemic. OP is a serious global health problem, with mounting patients are presenting under the aggravation of global population aging [4]. In China, the number of people suffering from osteoporosis is about 90 million, and the prevalence of osteoporosis in people over 50 years old is 19.2%, including 32.1% for women and 6.9% for men [5]. Thus, the management of patients with OP is extremely urgent. 

Up to now, medication still is the main treatment for OP, such as Alendronate, bisphosphonates, denousumab, and teriparatide [6]. Alendronate produced increased femoral neck and hip BMDs, reduced incidence of novel fractures, and lower incidence of serious adverse events, specifically those leading to study discontinuation [7]. Denosumab followed by alendronate and ibandronate had the highest influence on hip and femoral BMD [8]. Moreover, estrogen therapy also plays an important role in anti-osteoporosis [9]. Besides, several supplement therapies are recommended for preventing and treating OP, for example, intake of calcium and vitamin D, or taking some herbal medicine [10]. Although some medications have been shown to be effective, their side effects and high costs are another potential challenging issues [11]. Therefore, finding a drug that is effective and safe for osteoporosis is an important challenge for the industry. Additionally, careful planning for health and social services is the key to master management, including the use of drugs against osteoporosis and the early diagnosis of patients at risk [12].

As a complementary and adjunct therapy, Chinese herbal medicine characterized by fewer side effects and lower costs has been used to treat many diseases in China for long time [13, 14]. Radix Achyranthis Bidentatae (RAB), a kind of Chinese herbal medicine is known to regulate bone metabolism, promote bone formation and inhibit bone loss [15], which has a long history and a wide range of applications in Asia, particularly in China, Japan, and Korea for their effects on OP and bone fracture. Studies have shown that many ingredients of RAB have the effect of regulating glucose metabolism, Increasing blood flow, and anti-OP effects [16–18]. Inokosterone (IS), ecdysterone (ES), hyssop polysaccharide (HP), yssop saponin (YS) are the mainly active ingredients extracted from RAB by high performance liquid chromatography (HPLC). Several studies have demonstrated that multiple IRAB may possess anti-OP effects both in *vivo and vitro* [19]. Inokosterone (IS) can upregulate osteogenic differentiation-related genes and stimulate the formation of autophagosomes, which can ultimately promote differentiation of osteoblasts [20]. *β-*Edysterone can interfere in Dex-induced osteocyte apoptosis via activating of the PI3K/Akt signaling pathway in osteocytes [21]. However, RAB and its ingredients have not been applied in clinical practice due to scattered evidences and uncertain mechanisms. Therefore, we presented a systematic review and meta-analysis from the preclinical evidences of IRAB in animal models of OP to summarize the significant outcomes on efficacy and mechanisms.

## Methods

In this systematic review and meta-analysis, we followed PRISMA guidelines. There are no protocols preregistered for this review.

### Database and search strategies

We searched PubMed, EMBASE, Web of Science, Cochrane Library, Chinese National Knowledge Infrastructure, Wanfang, Chinese Biomedical Literature Database, as well as Chinese VIP databases for targeting articles published from inception to March 2023 in English or Chinese. Meanwhile, the references of selected articles were used as a Additional file 1. The retrieval strategy for PubMed was “Radix Achyranthis Bidentatae” AND “Osteoporosis” and was modified to suit other databases.

### Inclusion criteria

All randomized controlled animal trials were included if they met all of the following criteria: studies with (1) experimental groups received RAB or IRAB as monotherapy, while the corresponding control groups were treated with a blank treatment or received a placebo such as saline solution, (2) studies with conclusive results, and (3) animals models established using different methods, regardless of species, age, weight, or gender.

### Exclusion criteria

Exclusion criteria were as follows: (1) vitro studies, case reports, clinical trials, reviews, abstracts, comments, and editorials; (2) not animal OP model; (3) without control group;(4) compared with other traditional Chinese medicines; (5) lack of outcome indicator, and (6) duplicate publications.

### Outcome measurements

The primary outcome measures were bone mineral density of the femoral neck (F-BMD), serum calcium (Ca), serum phosphorus (P), serum alkaline phosphatase (ALP), bone gla protein (BGP), bone maximum stress (M-STRESS). The secondary outcome measure was the antiosteoporosis mechanisms of IRAB.

### Data extraction

Data were extracted by two authors (Haoran Zhu and Xiaxia Guo) independently, and the discrepancy was checked by the third investigator (Yinuo Fan). Data were also extracted from these studies independently by two investigators (Haoran Zhu and Xiaxia Guo) using a spreadsheet, including the author’s name (s), publishing date, animal species, age, gender, weight, sample size, OP modeling method, the use of anesthetics in the course of the experiment, the therapeutic regimen of the treatment and control groups, and primary and secondary outcomes and its intergroup differences. For continuous outcomes, we extracted the mean, standard deviation (SD), and participant number. If the study was involved in multiple intervention groups, we extracted data only for the group (s) involving IRAB and the control group (s). Author of these publications was contacted to obtain relevant data where necessary. The final results needed to be discussed with all the investigators to reach a consensus.

### Statistical analysis

*Data analyses were conducted* using RevMan 5.3 (Cochrane Collaboration, Oxford, United Kingdom) and STATA software (version 12.0 StataCorp, College Station, TX). A random-effects model was utilized for all analysis. Further subgroup study and sensitive analysis were performed to identify the possible cause of high heterogeneity (*I*^2^ > 50%). Moreover, Egger’s test was conducted to investigate the effect of publication bias. We calculated the pooled estimate as a standard mean difference (SMD) with a 95% confidence interval (CI) for continuous outcomes.

## Results

### Selection of studies

**Fig. 1:**
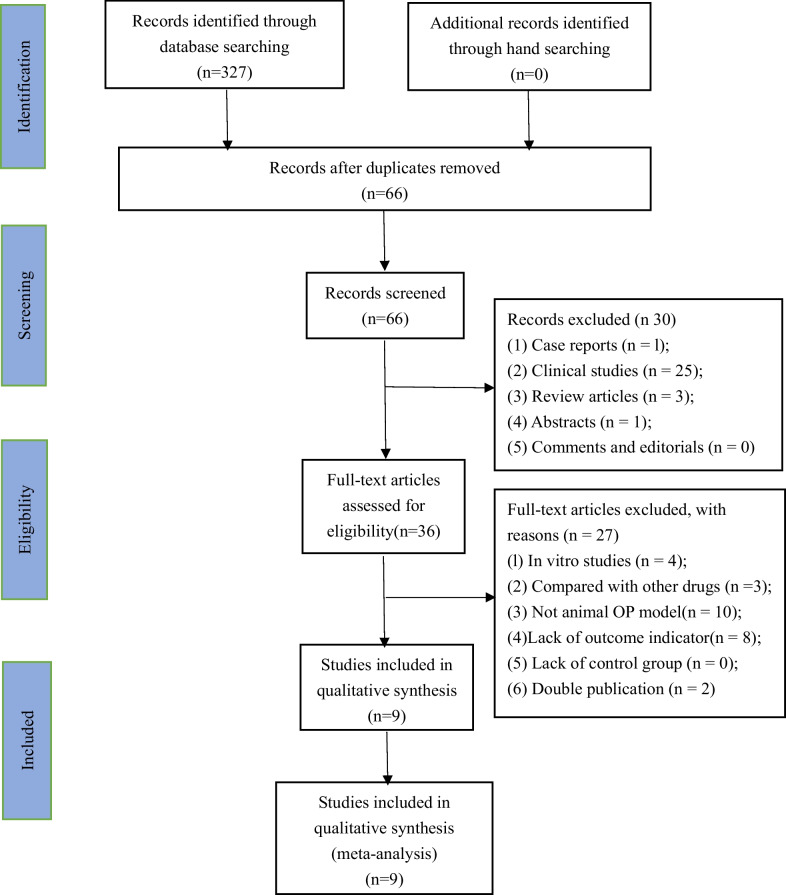
Flowchart of included studies

The detailed flow chart of literature identification and selection process is shown in Fig. 1. A total of 327 related articles were retrieved from eight databases. After removing duplicates, 66 studies remained by reading their titles and abstracts. We excluded 30 studies that are not related to this study after reading the titles and abstracts in detail. After reading the full text of the 36 remaining studies 27 articles were excluded for at least one of the exclusion criteria. Finally, 9 studies [22–30] were selected for this meta-analysis.

### General characteristics of the included studies

Characteristics of the 9 studies are summarized and shown in Table 1. All of them were published in English or Chinese between 2001 and 2021, which involved a total of 196 subjects with 98 in the experimental group vs 98 in the control group. Sprague–Dawley rats (SD) and Wistar rats were two different species utilized in 6 studies [22, 23, 25, 26, 28, 30] and 3 studies [24, 27, 29]. In a total of 9 studies, the weight of rats varied between 180 and 320 g, except for one study which did not report the weight of the rats. The animal sample size was ranged from 16 to 40, with the median sample size was 22 rats. The OP model was established by performing Bilateral oophorectomy or taking gavage of tretinoin (70 mg/ kg/d, qd) for 2 weeks. All the studies were administered by oral gavage with varied duration between 4 and 12 weeks. With regard to the specific IRAB, Radix Achyranthis Bidentatae was reported in three studies [22, 24, 30], Achyranthes Bdentata saponins in three studies [27–29], Achyranthan in one study [23], Achyranthes Bidentata Polysaccharides in one study [25], and Achyranthes Bidentata ecdystero in one study [26]. Nine studies used a dose gradient of IRAB by oral administration ranging from 14 mg/kg/d to 14 g/kg/d. In terms of primary outcome, F-BMD was evaluated in all studies [22–30], serum P, ALP, BGP in six studies [22, 24–26, 29, 30], serum Ca in seven studies [22–24, 26–29], and M-STRESS in five studies [22, 23, 25, 26, 30].

**Table 1:** Characteristics of the included 9 studies

Study (year)	Species (sex, n = experimental/control group, age)	Weight	Model (method)	Anesthetic	Experimental group	Control group	Outcome index	Intergroup/differences	Duration (weeks)
Wang Yan [22]	Female SD rats (10/10, NG)	NG	Bilateral oophorectomy was performed on rats	ketamine	By oral gavage of Radix Achyranthis Bidentatae (14g/(kg d)	By oral gavage of isometric NS	(1) BMD (F-BMD, L- BMD) (2) ELASTIC, M-STRESS, M-LORD, STIFFNESS (3) SerumCa, P, ALP, BGP	(1) P < 0.05 (2) P < 0.05 (3) P < 0.05	12
Lang Xiaoqin [23]	Female SD rats(20/20,10 months)	(280 ± 20)g	Bilateral oophorectomy was performed on rats	Chloral hydrate	By oral gavage of Achyranthan (400 mg/(kg d	By oral gavage of isometric NS	(1) BMD(F-BMD (2) ELASTIC, M-STRESS, M-LORD, STIFFNESS (3) SerumCa, TPACP5b, NTX, CTX (4) SerumOC, BAP (5) OPG, RANKL (6)BGP	(1) P < 0.05 (2) P < 0.05 (3) P < 0.05 (4) P < 0.05 (5) P < 0.05 (6) P < 0.05	12
Gao Changkun [24]	Male Wistar rats (10/10, NG)	150 g ± 20 g	By oral gavage of tretinoin (70 mg/ kg/d, qd) for 2 weeks	NG	By oral gavage of Radix Achyranthis Bidentatae (14mg/(kg·d)	By oral gavage of isometric NS	(1) BMD (F-BMD) (2) Serum Ca, P, ALP	(1) P < 0.05 (2) P < 0.05	4
Yang Hao [25]	Female SD rats (10/10, NG)	270 ~ 290 g	Bilateral oophorectomy was performed on rats	NG	By oral gavage of Achyranthes Bidentata Polysaccharides (8g/( kg· d)	By oral gavage of isometric NS	(1) BMD (F-BMD) (2) Serum, ALP, BGP, (3) Serum, NTx, TRAP (4) β-catenin, Runx2, mRNA, Osterix	(1) P < 0.05 (2) P < 0.05 (3) P < 0.05 (4) P < 0.05	12
Dong Qunwei [26]	Female SD rats (10/10,8 months)	267.18 ± 21.1 g	Bilateral oophorectomy was performed on rats	NG	By oral gavage of Achyranthes Bidentata ecdystero (4g/(kg d)	By oral gavage of isometric NS	(1) BMD (F-BMD, L- BMD) (2) ELASTIC, M-STRESS, M-LORD, STIFFNESS (3) SerumCa, P, ALP, BGP	(1) P < 0.05 (2) P < 0.05 (3) P < 0.05	4
Ren Xinci [27]	Male Wistar rats (8/8, NG)	180 ~ 220 g	By oral gavage of tretinoin (70 mg/ kg/d, qd) for 2 weeks	NG	By oral gavage of Achyranthes Bidentata saponins (300 mg/(kg· d)	By oral gavage of isometric NS	(1)BMD(F-BMD) (2) Serum Ca, P	(1) P < 0.05 (2) P < 0.05	4
Yang Guofu [28]	Female SD rats (10/10,6 months)	280 ~ 320 g	Bilateral oophorectomy was performed on rats	Pentobarbital sodium	By oral gavage of Achyranthes Bdentata saponins (300 g/(kg· d)	By oral gavage of isometric NS	(1)BMD(F-BMD, L- BMD) (2)Serum, BAP, BGP, TRAP NTx /Cr COLI	(1) P < 0.05 (2) P < 0.05	12
Ren Xinci [29]	Male Wistar rats (8/8, NG)	180 ~ 220 g	By oral gavage of tretinoin (70 mg/kg/d, qd) for 2 weeks	NG	By oral gavage of Achyranthes Bidentata saponins (300 mg/(kg· d)	By oral gavage of isometric NS	(1) BMD (F-BMD) (2) Serum ALP, Ca, P	(1) P < 0.05 (2) P < 0.05	4
Gao Weihu [30]	Female SD rats (12/12, 10 months)	280 ~ 320 g	Bilateral oophorectomy was performed on rats	Chloral hydrate	By oral gavage of Radix Achyranthis Bidentatae (1.5g/(kg d)	By oral gavage of isometric NS	(1) BMD (F-BMD) (2) M-STRESS (3) SerumCa, P, ALP BGP	(1) P < 0.05 (2) P < 0.05 (3) P < 0.05	12

### Risk of bias

Table 2 shows the risk of bias reported for each publication included in this meta-analysis. The risk of bias for each study was tested using the CAMARADES 10-item quality checklist, and the number of criteria met varied from 4/10 to 7/10 with the average of 5.44. All the included studies were peer-reviewed publications, two [27, 29] of them did not mention the control of temperature and the randomization. Seven [22–26, 28, 29] of them reported the ways of blinding induction of model. No study in this meta-analysis specially described sample-size calculations and allocation concealment, or reported exclusion criteria and outcomes of blinded assessment. Two studies [23, 28] did not declare Compliance with animal welfare regulations and the potential conflict of interests was not mentioned in three studies [22, 25, 30].

**Table 2:** Risk of bias of the included studies

Study	A	B	C	D	E	F	G	H	I	J	Total
Wang et al. [22]	√	√	√	√		√			√		6
Lang et al. [23]	√	√		√						√	4
Gao et al. [24]	√	√	√	√		√			√	√	7
Yang et al. [25]	√	√	√	√		√			√		6
Dong et al. [26]	√	√	√	√					√	√	6
Ren et al. [27]	√		√			√			√	√	5
Yang et al. [28]	√	√		√						√	4
Ren et al. [29]	√		√	√		√			√	√	6
Gao et al. [30]	√	√	√			√			√		5

Studies fulfilling the criteria of the following: A: peer-reviewed publication; B: control of temperature; C: random allocation to treatment or control; D:blinded induction of model (group randomly after modeling); E: blinded assessment of outcome; F: use of anesthetic without significant protective and toxic effects on bones; G: appropriate animal model (aged, hyperlipidemia, hypertensive, or diabetes); H: sample size calculation; I: compliance with animal welfare regulations (including three or more of the following points: preoperative anesthesia, postoperative analgesia, nutrition, disinfection, environment temperature, environment humidity, circadian rhythm, and euthanasia); J: statement of potential conflict of interests

### Effectiveness

#### F-BMD

**Fig. 2:**
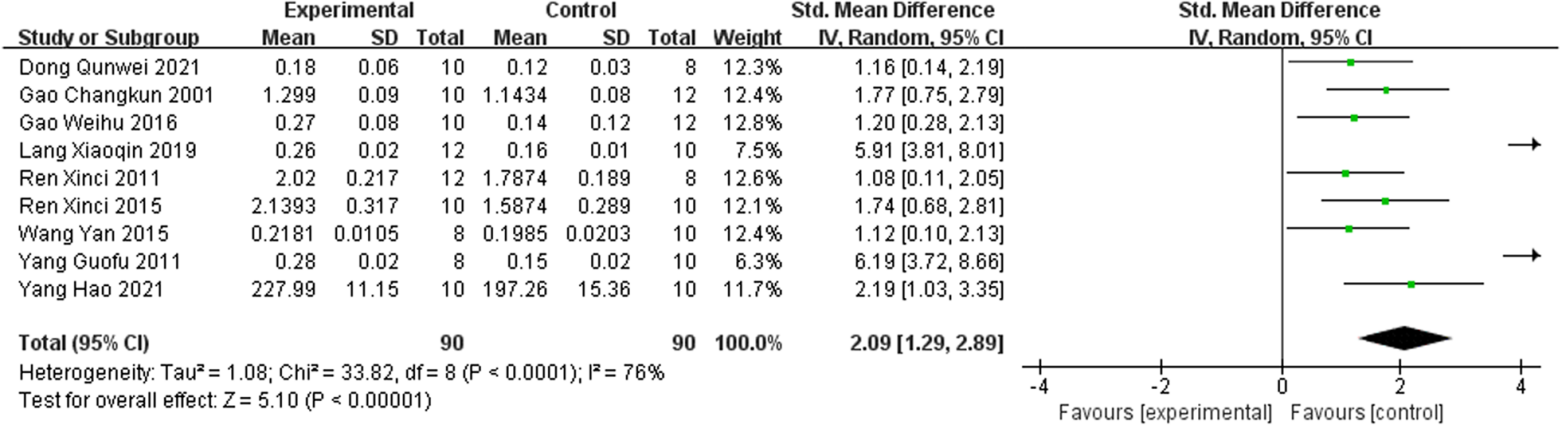
Forest plot of IFP versus control with regard to BMD-femur

As a primary outcome, all studies [22–30] reported on F-BMD and indicated that IRAB was significant for lifting BMD at the femur compared to the control group (SMD = 2.09; 95% CI = 1.29 to 2.89; heterogeneity Chi^2^ = 33.82, df = 8, P < 0.0001, *I*^2^ = 76% Fig. 2). The random-effect model was chosen given the significant heterogeneity among the included studies. Metaregression was not performed because only a small number of studies were included.

#### M-STRESS

**Fig. 3:**
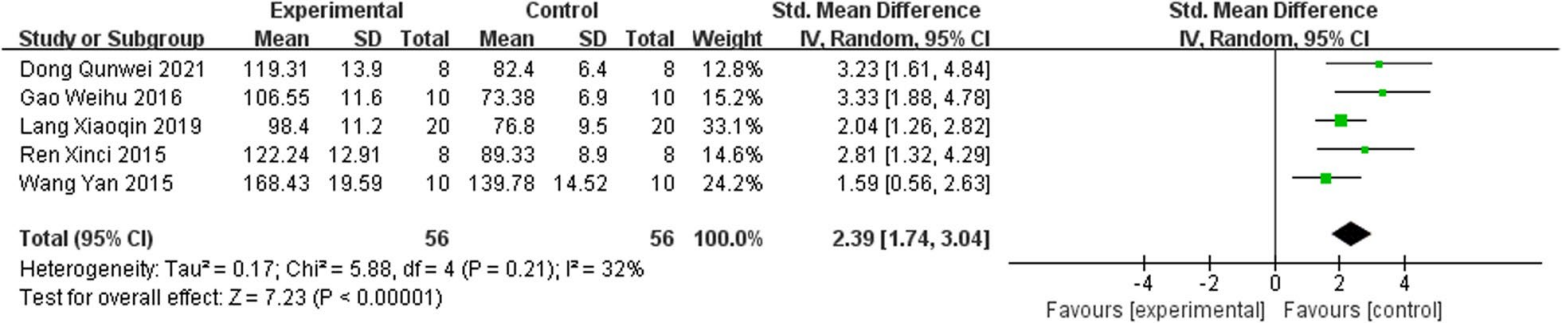
Forest plot of IFP versus control with regard to M-STRESS

Five studies [22, 23, 25, 26, 30]. reported IRAB versus the control group according to M-STRESS The pooled results indicated that IRAB was significant for raising M-STRESS compared to the control group (SMD = 2.39; 95% CI = 1.74–3.04; heterogeneity Chi^2^ = 5.88, df = 4, *I*^2^ = 32%, *P* = 0.21; Fig. 3).

#### Serum calcium

**Fig. 4:**
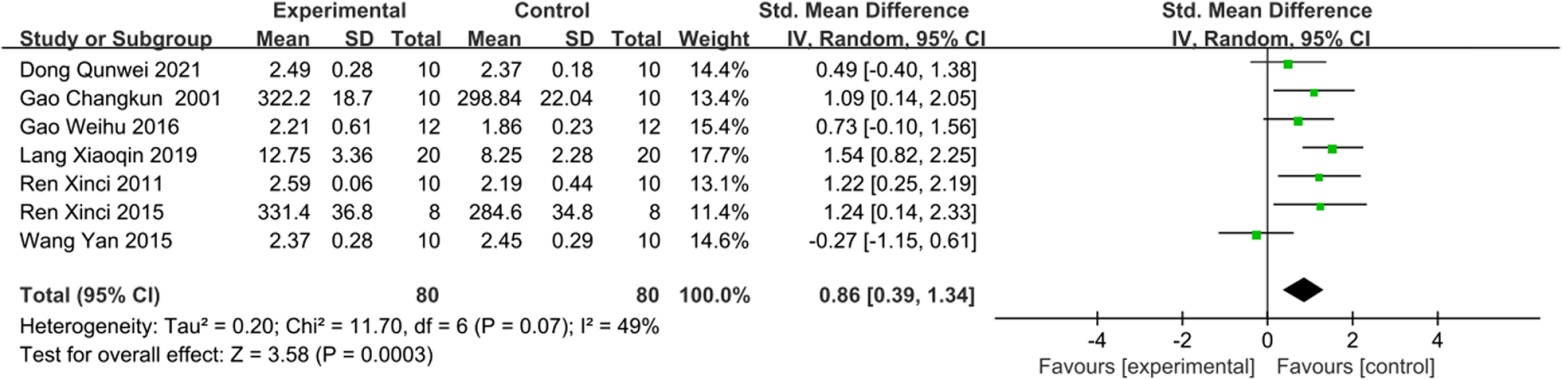
Forest plot of IFP versus control with regard to Serum Calcium

Seven studies [22–24, 26–29] examine the serum calcium and six results indicated that serum calcium was significantly increased after IRAB treatment compared with control group (SMD = 0.86; 95% CI = 0.39–1.34; heterogeneity Chi^2^ = 11.7, df = 6, *I*^2^ = 49%, *P* = 0.07; Fig. 4).

#### Serum phosphorus

**Fig. 5:**
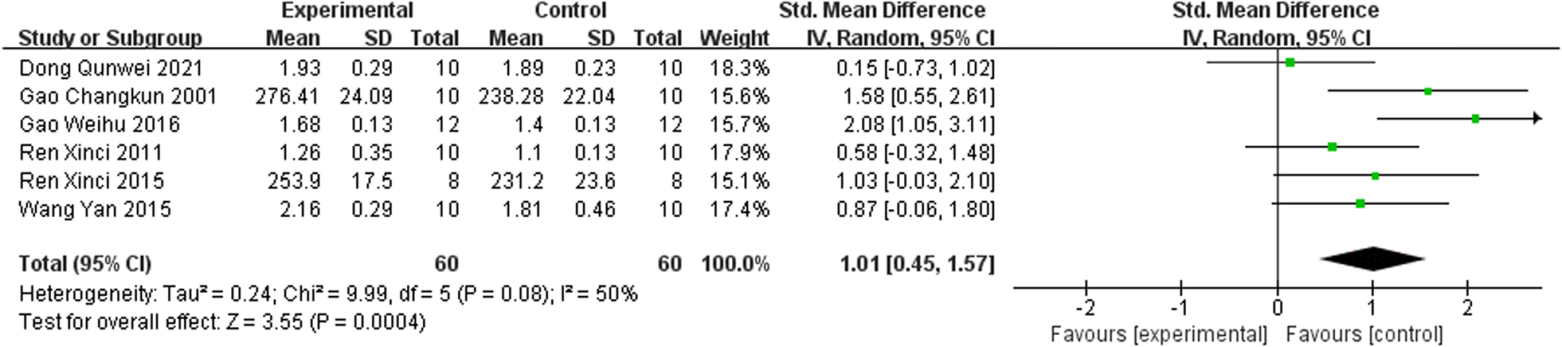
Forest plot of IFP versus control with regard to Serum Phosphorus

There were six studies [22, 24–26, 29, 30] comparing IRAB with the control group about P. The pooled results indicated that IRAB significantly increased P compared to the control group (SMD = 1.01; 95% CI = 0.45–4.57; heterogeneity Chi^2^ = 9.99, *I*^2^ = 50%, P = 0. 08; Fig. 5).

#### Serum alkaline phosphorus

**Fig. 6:**
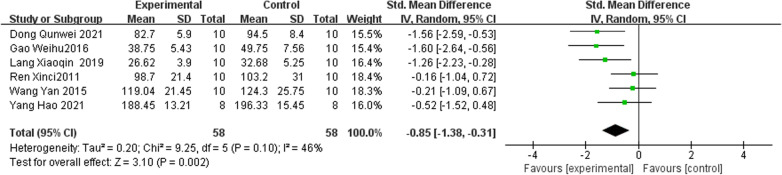
Forest plot of IFP versus control with regard to Serum Alkaline Phosphorus

Six studies [22, 24–26, 29, 30] results revealed that the level of ALP was significantly reduced after a treatment of IRAB compared with the control group [31] (SMD =  − 0.85; 95% CI =  − 1.38 to − 0.31; heterogeneity Chi^2^ = 9.25, *I*^2^ = 46%, P = 0.10; Fig. 6).

#### Bone Gla protein

**Fig. 7:**
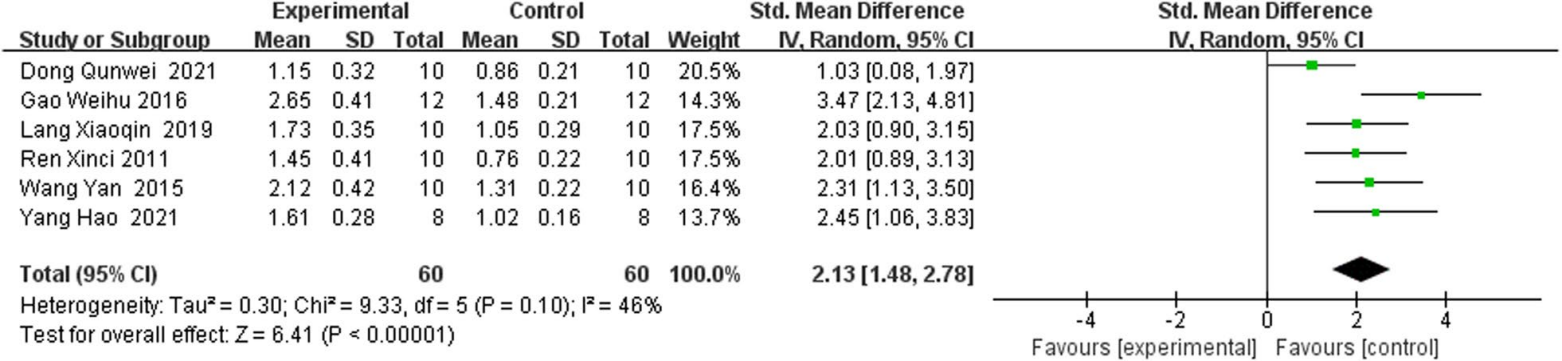
Forest plot of IFP versus control with regard to Bone Gla Protein

Six studies [22, 24–26, 29, 30] reported IRAB versus the control group according to BGP [25, 28]. The pooled results indicated that IRAB was significant for decreasing BGP compared to the control group (SMD = 2.13; 95% CI = 1.48–2.78; heterogeneity Chi^2^ = 9.33, df = 5, *I*^2^ = 46%, *P* = 0.10; Fig. 7).

### Subgroup analysis

**Fig. 8:**
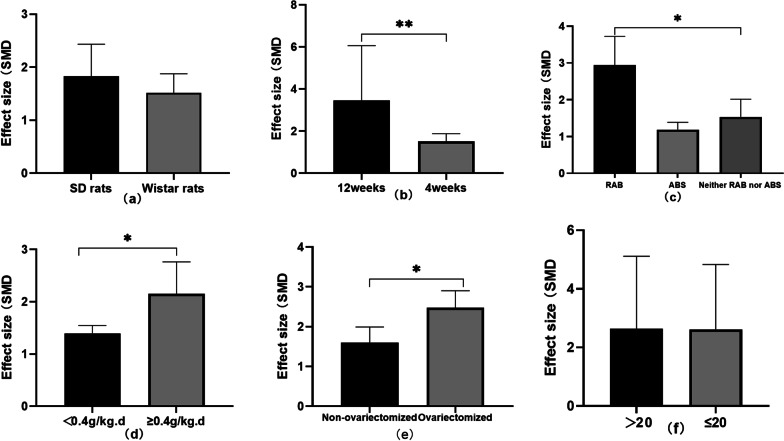
Subgroup analyses of the BMD-femur. **a** The different effect sizes between the SD rat group and the Wistar rat group. **b** The different effect sizes between the 12 weeks group and 4 weeks group. **c** The different effect sizes between the different kind of RAB group. **d** The different effect sizes between different dosage groups. **e** The different effect sizes between the ovariectomized model group and the No-ovariectomized model group. **f** The different effect sizes between different sample size groups. *P < 0.05 between subgroups

There are six different potential confounding factors (including animal species, duration, kind of IRAB, dosages of IRAB, model methods, and sample size) that may increase the heterogeneity of outcome measures were explored using subgroup analysis of BMD-femur No difference in the effective size was found between the SD rat group [22, 23, 25, 26, 28, 30] and the Wistar rat group [24, 27, 29] in the subgroup analysis of animal species (SMD = 2.23 ± 1.85 versus SMD = 1.49 ± 1.02, respectively, *P* = 0.439, Fig. 8a and heterogeneity of both groups did not decrease obviously. In the subgroup analysis of duration, the 12 weeks duration group [22, 23, 25, 28, 30] showed better effective size than the 4 weeks duration group [24, 26, 27, 29] (SMD = 2.29 ± 1.21 versus SMD = 1.49 ± 1.02, respectively, *P* = 0.0087, Fig. 8b with significantly reduced heterogeneity of both groups. In the subgroup analysis of kind of IRAB, significant difference was found between the three groups (RAB-SMD = 2.93 ± 1.21 versus ABS-SMD = 1.19 ± 0.890 versus Neither RAB nor ABS-SMD = 1.53 ± 0.963, respectively, RAB vs ABS P = 0.0181, RAB vs Neither RAB nor ABS p = 0.045, ABS vs Neither RAB nor ABS *P* = 0.729, Fig. 8c) and the heterogeneity of the three groups decreased substantially. Furthermore, the high-dosage IRAB group [22, 24, 26, 28, 30] (≥ 0.4 g/kg, qd) showed greater effect size than in the low-dosage IRAB group [23, 25, 27, 29] (< 0.4 g/kg, qd) (SMD = 2.29 ± 1.21 versus SMD = 1.49 ± 1.02, respectively, *P* = 0.045, Fig. 8d, and heterogeneity of two groups reduced substantially. Besides in the subgroup analysis of modeling methods, the ovariectomized model group [24, 27, 29] showed better effect size than the nonovariectomized model group [22, 23, 25, 26, 28, 30] (SMD = 1.58 ± 0.81 versus SMD = 2.06 ± 1.12, respectively, P = 0.027, Fig. 8e) with significantly reduced heterogeneity of both groups. However, no difference was shown between the high-sample group [23, 27, 29, 30] (> 20) and the low-sample group [22, 24–26, 28] (≤ 20) (SMD = 2.00 ± 1.02 versus SMD = 1.82 ± 0.82, respectively, P = 0.99).

### Publication bias and sensitivity analysis

**Fig. 9:**
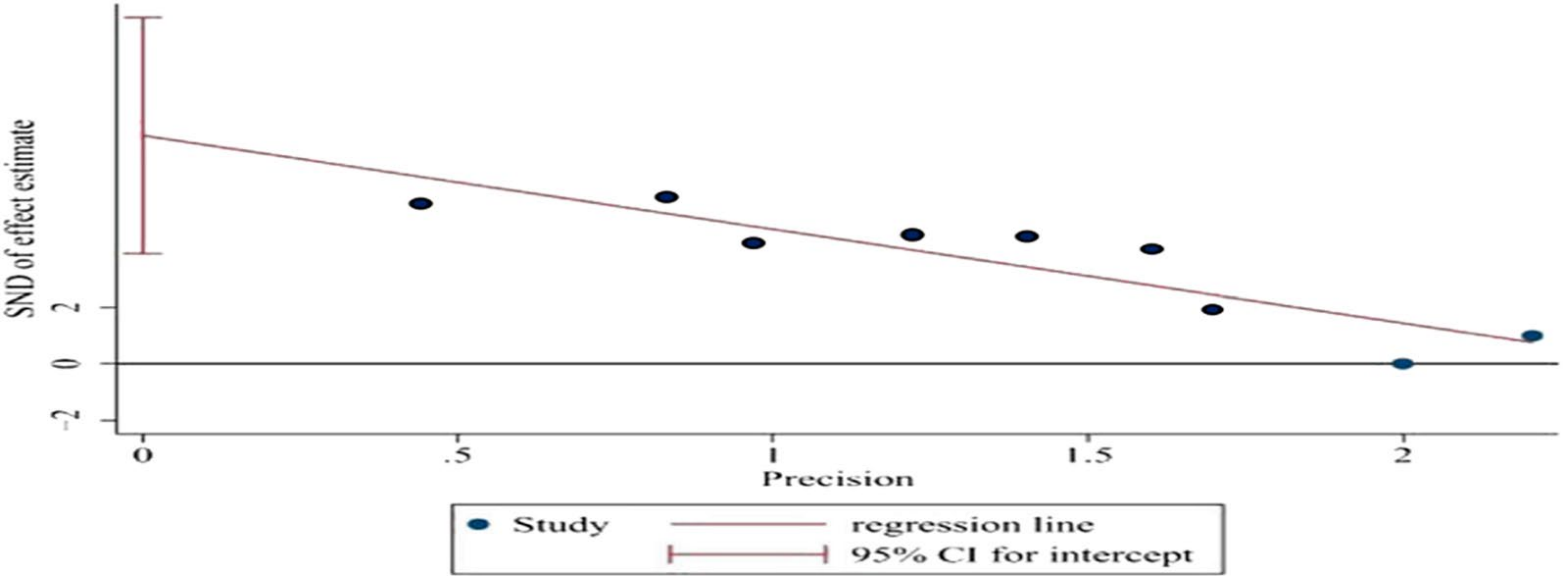
Egger’s test of BMD-femur indicated that there was publication bias. *p* = 0.002, *t* = 5.14

**Fig. 10:**
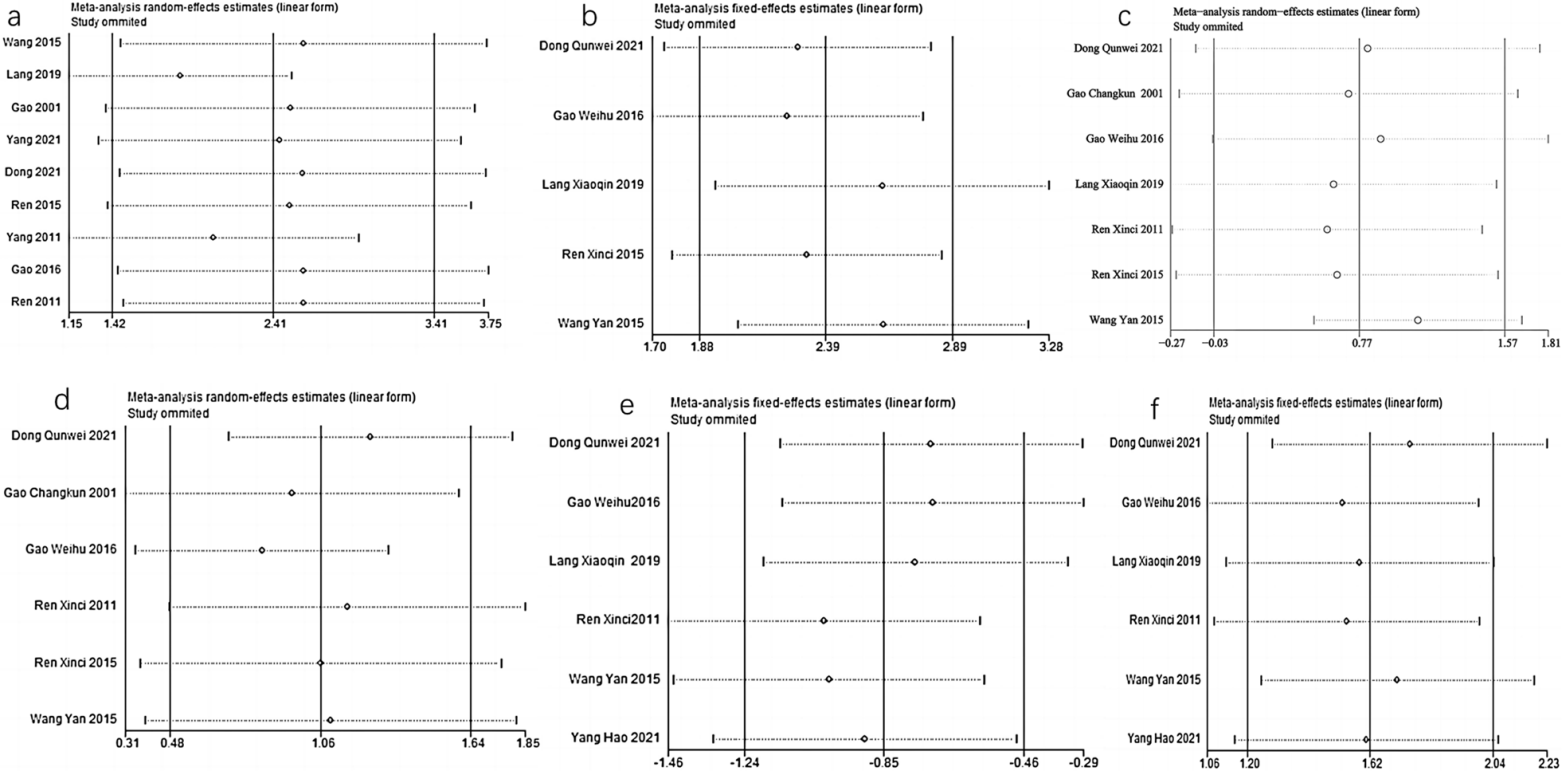
Sensitivity analysis. **a**–**f** represent the sensitivity of BMD-femur, M-STRESS, Serum-Ca, Serum-P, ALP, BGP, respectively. The sensitivity analysis was conducted by omitting single studies one by one, and no study with critical influence was found

Egger’s test was applied to assess the potential publication bias in this meta-analysis and identified several publication biases (BMD-femur, *p* = 0.002, Fig. 9). Sensitivity analysis were also conducted by omitting each study, and no obvious effect was found (Fig. 10).

## Discussion

### Summary of evidence

To the best of our knowledge, this is the first preclinical systematic review and meta-analysis to estimate the efficacy and possible mechanism of IRAB for the OP animal model. Nine high-quality RCTs involving 196 rats with the OP model were enrolled in the analysis. The results illustrated that IRAB could significantly increase BMD, Ca, P, BGP, while ALP was decreased remarkably by IRAB in OP animal models. Moreover, IRAB could significantly improve the bone biomechanical indicator bone maximum load and elasticity modulus. Therefore, the findings indicated that RAB is a positive anti-OP drug though multiply mechanism. However, we discovered the outcome F-BMD presented high heterogeneity in this meta-analysis. Duration, kind of IRAB, dosages of IRAB, model methods, and sample size were the mainly resource of high heterogeneity though subgroup analysis. Thus, more high-quality studies involving large sample sizes should be conducted to confirm our finding.

### Strengths

As far as we know, this is the first meta-analysis to report effect and mechanism of RAB in anti-OP on animal model. All the enrolled studies were RCT trials, including nine trials with 196rats. Moreover, two registers separately evaluated the entry data components and the quality control appraisal of all the data, in order to reduce bias. Additionally, subgroup analysis was performed to identify the origin of heterogeneity.

Consequently, no publication bias was reported in this meta-analysis, and sensitivity estimation revealed that the findings of this meta-analysis are comparatively robust.

### Limitations

Nonetheless, this study has several limitations. Firstly, although we researched eight databases without any restriction on language, it is possible that we may have omitted some relevant studies. Secondly, selection bias was unavoidable because negative outcomes are not always reported or published. Thirdly, high heterogeneity of F-Bone influenced could compromise the viability of our findings despite subgroup analysis were done. Fourthly, the OP modeling methods, the specific kind of IRAB, dosage of IRAB, administration approaches, and period of IRAB treatments differed remarkably in the included studies. Finally, most of the included studies in the meta-analysis were conducted in China, this also will jeopardize the validity of results.

### Implications

Animal experiments are an important cornerstone in translating experimental results into clinical treatments for human diseases [32]. Different animal models were designed to research the pathophysiology and treatments of OP, including two mainly models: models with increased bone resorption and models with reduced bone formation. This study comprehensively includes the ovariectomized OP model and nonovariectomized OP model to evaluate the efficacy and mechanisms of IRAB for OP. Ovariectomized animal models are widely used to study postmenopausal osteoporosis because it reduces estrogen levels and induces bone loss. Estrogen plays a very important role in bone reconstruction. Estrogen stimulates osteoclasts to secrete osteoprotegerin, insulin-like growth factors, and transforming growth factors. Enhanced bone resorption due to down-regulation of the levels of these cytokines when estrogen is insufficient [33]. The results of subgroup analysis suggested that the ovariectomized OP model group showed better effect size than the nonovariectomized OP model group in regard to BMD-femur (SMD = 1.58 ± 0.81 versus SMD = 2.06 ± 1.12, respectively, *P* = 0.027, Fig. 8e), which suggests that the different OP model methods may be the source of high heterogeneity. Thus, we suggest an ovariectomized OP model be adopted to assess OP in future studies.

BMD is an important sign of bone quality, which is of great significance in medicine [34]. The results showed that the BMD value of the experimental group increased significantly, indicating that RAB or its IRAB could promote bone formation. Inokosterone (IS) is one of the most major ingredients of RAB and was also the most used IRAB in the included studies because it has the highest content in RAB and is easy to extract from RAB. Inokosterone (IS) significantly up-regulated the expression levels of Collagen I, OPN, OPG and OCNmRNA genes related to osteogenic differentiation. Edysterone is another furanocoumarin compound of RAB and is derived from RAB*.* Edysterone can interfere in Dex-induced osteocyte apoptosis via activating of the PI3K/Akt signaling pathway in osteocytes. Ecdysterone can significantly promote the proliferation and differentiation of osteoblasts, while up-regulating ERα, β-catenin and down-regulating pAMPKα. Other IRAB including hyssop polysaccharide (HP), yssop saponin (YS) were also reported in our included studies, however, the number of studies was relatively less and their effect size was lower than Inokosterone (IS) and ecdysterone (ES). Therefore, Inokosterone (IS) and ecdysterone (ES) may be recommended as potential candidates of anti-OP drugs in the future studies. Additionally, BGP is mainly synthesized by osteoblasts and can bind to bone matrix to maintain the normal rate of bone mineralization. The level of BGP in serum can reflect the activity of osteoblasts [35, 36]. The results in this meta-analysis revealed that IRAB was significant for decreasing BGP compared to the control Group (SMD = 2.13; 95% CI = 1.48 to 2.78; heterogeneity Chi^2^ = 9.33, df = 5, *I*^2^ = 46% *P* = 0.10; Fig. 7). Besides, bone ALP is mainly synthesized by osteoblasts and released into the blood. The main function of ALP is to promote bone formation, and the serum ALP level can partially reflect the active degree of bone formation. Six studies results revealed that the level of ALP was significantly reduced after a treatment of IRAB compared with the control group (SMD = − 0.85; 95% CI =  − 1.38 to − 0.31; heterogeneity Chi^2^ = 9.25, *I*^2^ = 46%, *P* = 0.10; Fig. 6).

### Possible mechanisms

**Fig. 11:**
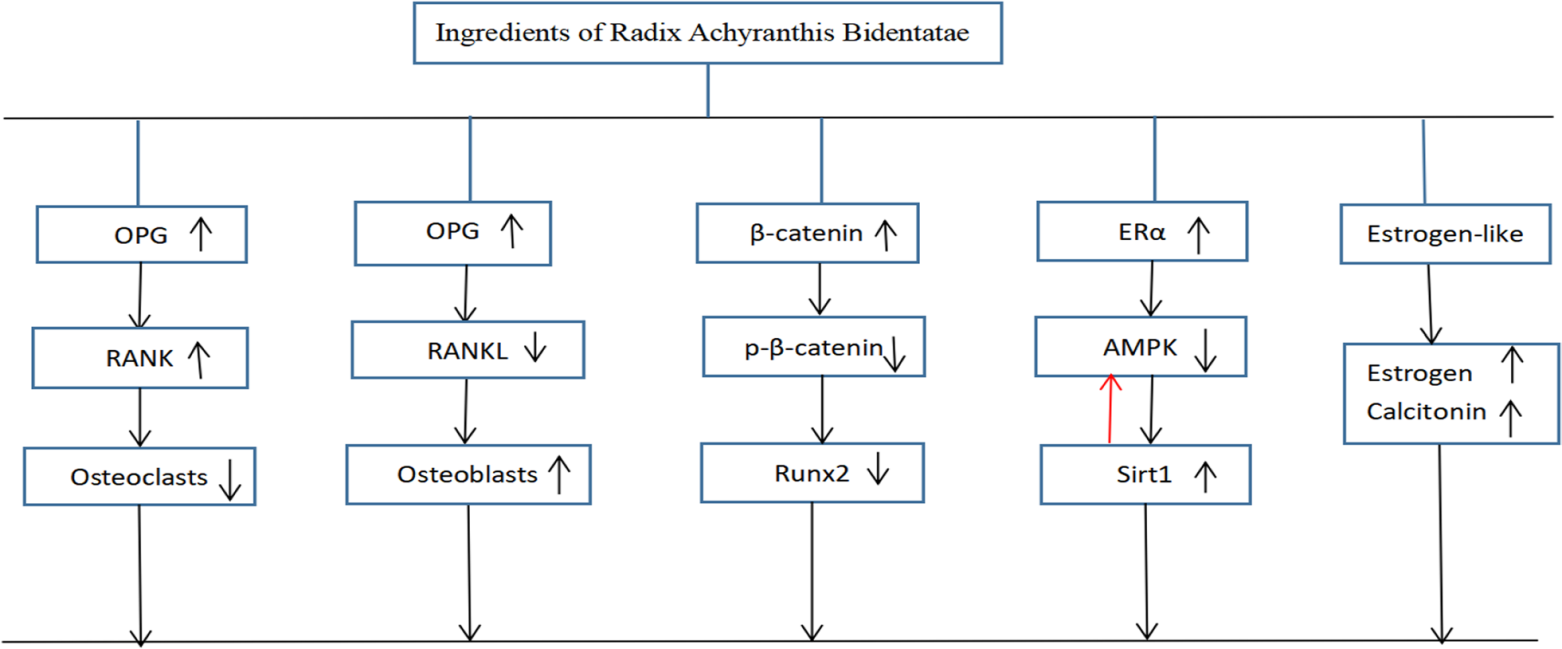
A schematic representation of antiosteoporosis mechanisms of IRAB

The possible mechanisms of IRAB that mediated anti-OP effects in the included studies are summed up as follows:(1) Osteoprotegerin/receptor activator of nuclear factor-κB ligand/receptor activator of nuclear factor-κB (OPG/RANKL/RANK) signal pathway: IRAB could highly increase OPG secretion and competitively binds RANK, thereby inhibiting RANK/RANKL-mediated bone resorption activity, promoting bone formation and exerting osteoprotective effect [37]. (2) Wnt/β-catenin/Runx2 signal pathway: IRAB could activate the Wnt/β-catenin signaling pathway, upregulate β-catenin, cytosolic β-catenin expression, and downregulate p-β-catenin expression, thereby promoting expression of Runx2 and Osterix to decrease [38, 39]. (3) ERα- AMPK-Sirt1 signal pathway: IRAB can promote the proliferation of primary osteoblasts as well as the division, meanwhile, increase the expression of ERα and inhibit the expression of AMPK. However, the expression of AMPK was found to be up-regulated after silencing ERα by siRNA tended to be upregulated. AMPK and Sirt1 are able to feed back to each other regulation, AMPK activates Sirt1, and Sirt1 in turn activates AMPK [40]. Besides, studies reported that if AMPK is inhibited, the number of apoptotic osteoblasts would increase, and further silenced AMPKα, the apoptotic effect of osteoblasts would be more obvious, as seen Sirt1/AMPK activation can reduce the apoptosis of osteoblasts [41]. (4) Estrogen-like effect: IRAB had the similar effect of phytoestrogen on inhibiting bone resorption by participating in the binding of estrogen receptor. On the one hand, IRAB may promote the synthesis and secretion of estrogen outside the ovary [42, 43]. On the other hand, it may enhance the secretion of thyroid calcitonin to exert it anti-OP effect [44]. The mechanism diagram is summarized in Fig. 11.

## Conclusion

This preclinical systematic review provided preliminary evidence that IFP was capable of partially exerting anti-OP effects in animal models probably through the OPG/RANKL/RANK, Wnt/β-catenin/Runx2, ERα-AMPK-Sirt1, Estrogen-like effect signaling pathway. Taken together, the findings suggest the possibility of developing IRAB as a drug for the clinical treatment of OP.

## Supplementary Information

**Additional file 1. Supplementary Table**. A detailed list of excluded studies with reasons, references and number.

## Data Availability

The datasets used and/or analyzed during the current study are available from the corresponding author on reasonable request.

## Abbreviations

OP: Osteoporosis
RAB: Radix Achyranthis Bidentatae
IRAB: Ingredients of Radix Achyranthis Bidentatae
DXM: Dexamethasone
F-BMD: Bone mineral density of the femoral neck
Ca: Serum calcium
P: Serum phosphorus
ALP: Serum alkaline phosphatase
BGP: Bone gla protein
M-STRESS: Bone maximum stress
IS: Inokosterone
ES: Ecdysterone
HP: Hyssop polysaccharide
YS: Yssop saponin
SD: Sprague–Dawley
NS: Normal saline
BMD: Bone mineral density
Runx2: Runt-related transcription factor 2
AKT: Protein kinase B
RANKL: Receptor activator of nuclear factor-κ B ligand
OPG: Osteoprotegerin
