# Supplementary material for: Antiosteoporosis effect and possible mechanisms of the ingredients of Radix Achyranthis Bidentatae in animal models of osteoporosis: systematic review and meta-analysis of in vivo studies
Source: J Orthop Surg Res. 2023 Jul 26;18:531. doi: 10.1186/s13018-023-04031-w (PMC10369767; doi:10.1186/s13018-023-04031-w)
Supplement: Supplementary file 1 — Additional file 1. Supplementary Table. A detailed list of excluded studies with reasons, references and number. [file 13018_2023_4031_MOESM1_ESM.docx]

Stable:A detailed list of excluded studies with reasons,references and number

| Reasons | References | Number |
| --- | --- | --- |
| Case reports | Cai Quanju. A case report of adverse reactions caused by aconite herbal medicine[J]. Journal of Qinghai Medicine,2016,46(06):64. | 1 |
| Clinical studies | Yan Xianghua. Clinical study on the treatment of postmenopausal osteoporosis with cow knee and papaya soup for liver and kidney yin deficiency type [D]. Hebei North College, 2021. | 25 |
|  | Li Junhao. Evaluating the effect of optimal Niubizi-Eucommia combination to intervene in glucocorticoid osteoporosis based on homogeneous design[D]. Shanghai University of Traditional Chinese Medicine,2019. |  |
|  | WANG S, ZENG M, LI B, et al. Raw and salt-processed Achyranthes bidentata attenuate LPS-induced acute kidney injury by inhibiting ROS and apoptosis via an estrogen-like pathway [J]. Biomed Pharmacother, 2020, 129: 110403. |  |
|  | WENG X, LIN P, LIU F, et al. Achyranthes bidentata polysaccharides activate the Wnt/beta-catenin signaling pathway to promote chondrocyte proliferation [J]. Int J Mol Med, 2014, 34(4): 1045-50. |  |
|  | DAYUAN Z, LAN L, HUI C, et al. Study on Computer Screening and Drug Properties of Herbs Intervening in Copper Death [J]. Comput Math Methods Med, 2023, 2023: 3311834. |  |
|  | LI L, WANG B, ZHANG T, et al. [Optimization of Ethanol Extraction and  Water Extraction Technologies of Zuogui Pills by Orthogonal Test] [J].  Zhong Yao Cai, 2015, 38(8): 1724-7. |  |
|  | Du Yuanwon, Chen Qianhu, Jia Shaohui, Sun Daming, Wang Xuehong. Experimental study on the combined treatment of osteoporotic fractures with the combination of niu-knee and bamboo ginsenoside and juniperidol diglucoside[J]. Chinese Journal of Traditional Chinese Medicine Orthopedics and Traumatology,2015,23(06):9-13. |  |
|  | Cai F, Qiu Y, Zhao LJ. Progress of experimental research on the treatment of osteoporosis with single herbal medicine to activate blood circulation and remove blood stasis[J]. Journal of Traditional Chinese Medicine,2014,20(12):55-57. |  |
|  | Tian Shuo,Miao Mingsan. Exploration on the chemical, pharmacological and application characteristics of Niubizi[J]. Journal of Traditional Chinese Medicine,2014,29(08):1186-1188. |  |
|  | Lu Qingxiu. Study on the disassembly and pharmacological effects of the effective parts of Huai Niu Knee for kidney and bone [D]. Heilongjiang University of Traditional Chinese Medicine,2014. |  |
|  | Bian Yuqun. Theoretical and experimental study on the treatment of primary osteoporosis by kidney tonic method[D]. Nanjing University of Traditional Chinese Medicine,2014. |  |
|  | Huang Pui-tze, Ma Yong. Exploration of the rules of formula combination in the treatment of primary osteoporosis in Chinese medicine[J]. World Journal of Integrated Chinese and Western Medicine,2012,7(11):926-928. |  |
|  | Bian Yuqun, Li Chao, Tan Feng, Fan Qiaoling. A review of recent experimental studies on kidney tonic formulae for osteoporosis[J]. Journal of Shanxi College of Traditional Chinese Medicine,2012,13(03):141-143+162. |  |
|  | Zheng LY, Huang HUijuan. The formula of Zuo Gui Wan for the treatment of osteoporosis and its pharmacological study[J]. China Health Industry,2012,9(15):152+154. |  |
|  | Tang JF, Lu YE, Hu XQ. Treatment of 30 cases of osteoporosis in the elderly with the addition of Ubiquitous Knee Formula[J]. Modern Chinese medicine,2011,31(04):24-25. |  |
|  | He Jianeng, Wang Changxing, Dong Liqiang, Shen Jianguo, Jiang Tao, Yin Hang, Dong Xueliang, Liu Qi. The understanding of osteoporotic fractures in Chinese medicine and the study of single herbal medicine on the healing of osteoporotic fractures[J]. Chinese Journal of Traditional Chinese Medicine and Orthopedics,2010,18(07):67-69. |  |
|  | Xie Jihui,Li Lijuan. Treatment of 36 cases of osteoporotic thoracolumbar compression fractures with Lugulan polypeptide injection combined with kidney-boosting Chinese herbal medicine[J]. Contemporary Medicine,2009,15(07):137. |  |
|  | Triterpenoid extracts of Boswellia serrata and their application in anti-osteoporosis drugs. Jiangsu Province, Nanjing University, 2009-01-01. |  |
|  | Zhang Shanshan,Guo Baorong,Zhang Guo Li. Clinical observation on the treatment of diabetic osteoporosis in the elderly with Yi kidney and strong bone combination[J]. Journal of Shandong University of Traditional Chinese Medicine,2007(03):204-206. |  |
|  | Anti-osteoporosis effect of Boswellia serrata. Jiangsu Province, Nanjing University, 2007-01-01. |  |
|  | Luo M,Xu ZW. Observation of 120 cases of osteoporotic low back pain treated with the method of tonifying kidney and activating blood[J]. Journal of Practical Chinese Medicine,2006(08):463. |  |
|  | DU Rongbing,CHEN Bin. Clinical observation on the treatment of osteoarthritis of the knee with liver and kidney deficiency by combining local closure with Du Teng Fang[J]. Shanxi Traditional Chinese Medicine,2023,39(02):19-21 |  |
|  | Wei M, Gu CHM, Wang YX, Guan YH. Oxysterone mediates Notch pathway to affect the osteogenic effect of dental osteoblasts[J]. Journal of Microcirculation,2022,32(04):9-14. |  |
|  | Zhao Y,Li N,Peng XY. Effectiveness of Cistanches and Oxalis soup plus flavor combined with alendronate sodium in the treatment of osteoporosis and its effect on fracture incidence[J]. Journal of Practical Hospital Clinics,2022,19(05):36-40. |  |
|  | Yan Xianghua. Clinical study on the treatment of postmenopausal osteoporosis with cow knee and papaya soup for liver and kidney yin deficiency type [D]. Hebei North College, 2021. |  |
| Review articles | LU Weida, REN J, ZHANG Zhihong, ZHU Yuhui, LI Bingqi, GUO Hai-ling. Research progress of single herbal medicine in the treatment of osteoporosis and osteoarthritis[J].Henan TCM,2021,41(03):478-482. | 3 |
|  | WANG J, FENG J, DENG S, et al. Network Pharmacology Analysis of the Effects of Achyranthis Bidentatae Radix Plus Semen Vaccariae on Migraine-induced Erectile Dysfunction [J]. Combinatorial chemistry & high throughput screening, 2022, 25(9): 1474-87. |  |
|  | WU L, HAO Y, DAI C, et al. Network Pharmacological Study of Achyranthis bidentatae Radix Effect on Bone Trauma [J]. Biomed Res Int, 2021, 2021: 5692039. |  |
| Abstracts | Wang Shu, Gao Yi, Sun Fenglei, Shi Wei. Mechanistic study on the treatment of osteoporosis with single herbal medicine[J]. World abstract of latest medical information,2018,18(86):80-81. | 1 |
| In vitro studies | Chen H-T, Luo Y-W, Chen D-F, Xu L-L, Liu Y-M, Wang B, Xie P-J. Study on migration and CXCR4 expression of rat bone marrow mesenchymal stem cells stimulated by bovine knee cuprostanolone in vitro[J]. Chinese Journal of Osteoporosis,2019,25(11):1550-1555. | 4 |
|  | Li Chen-Chun. The effect of niuqin polysaccharide on the expression levels of Fadd, Beclin-1 and LC3II/Ⅰ in human KOA chondrocytes[D]. Hunan University of Traditional Chinese Medicine,2019. |  |
|  | Huang Yongqing. Study on the effect of oxysterone on the proliferation and differentiation of rat primary osteoblasts and mouse osteoblast cell line MC3T3-E1[D]. Guangzhou University of Traditional Chinese Medicine,2019. |  |
|  | WEI Yuanji,LI Junhao,WANG Libo,WANG Chenglong,DAI Weiwei. The active ingredient β-ecdysterone of cowberry interferes with dexamethasone-induced osteoclast apoptosis via Akt signaling[J]. Chinese Journal of Osteoporosis,2019,25(03):375-379. |  |
| Compared with other drugs | DAI Jinyang,LI Jia,XIAO Yuanmei,HU Jiaqi,LU Jiaxing,SUN Yantao. Effects of He Shou Wu and Huai Niu Knee on osteoporosis in mice before and after their combination[J]. Strait Pharmacology,2021,33(11):36-38. | 3 |
|  | Zhang Yu, Zhang Sun Zhengyuan, Wang Libo, Wang Chenglong, Cai Juefeng, Dai Weiwei. Study on the intervention of glucocorticoid osteoporosis model in mice with the combination of cowberry and eucommia[J]. Chinese Journal of Osteoporosis,2022,28(05):643-647. |  |
|  | Gao Weihui, Wu Fenfen, Duan Xiaoqing, Liu Yun, Deng Guiming, Lin Wang Senzhi. Experimental study on the effects of Eucommia-cowberry medicine on estradiol and bone mineral density in intervening de-ovalized osteoporotic rats[J]. Zhongnan Pharmacology,2016,14(08):820-823. |  |
| Not animal OP model | Ma Dujun,Peng Liping,Jiang Shunwan et al. Effects of BMSC-Exos intervention with alcoholic extract of Boswellia serrata on local bone tissue ultrastructure and inflammatory vesicles in rabbits with OA model[J]. Journal of Traditional Chinese Medicine,2022,28(01):12-18. | 10 |
|  | TAO Yi, JIANG Enchi, JIANG Huijie, YAN Jizhong, CAI Baochang. Effects of concocted products of Boswellia serrata on osteoporotic zebrafish[J].Journal of Zhejiang University of Technology,2020,48(05):504-507+525. |  |
|  | Wei Yuanji. An orthogonal design-based investigation of the effect and mechanism of the combination of active ingredients of Niubizi-Eucommia cuspidata to intervene in a zebrafish model of glucocorticoid osteoporosis [D]. Shanghai University of Traditional Chinese Medicine,2019. |  |
|  | ZHOU X, SIU W S, ZHANG C, et al. Whole extracts of Radix Achyranthis Bidentatae and Radix Cyathulae promote angiogenesis in human umbilical vein endothelial cells in vitro and in zebrafish in vivo [J]. Exp Ther Med, 2017, 13(3): 1032-8. |  |
|  | WU J, LI J, LI W, et al. Achyranthis bidentatae radix enhanced articular distribution and anti-inflammatory effect of berberine in Sanmiao Wan using an acute gouty arthritis rat model [J]. J Ethnopharmacol, 2018, 221: 100-8. |  |
|  | WANG J S, FENG J L, DAI H H, et al. Potential mechanism of Achyranthis bidentatae radix plus semen vaccariae granules in the treatment of diabetes mellitus-induced erectile dysfunction in rats utilizing combined experimental model and network pharmacology [J]. Pharmaceutical biology, 2021, 59(1): 547-56. |  |
|  | LEE J H, WEI Y J, ZHOU Z Y, et al. Efficacy of the herbal pair, Radix Achyranthis Bidentatae and Eucommiae Cortex, in preventing glucocorticoid-induced osteoporosis in the zebrafish model [J]. J Integr Med, 2022, 20(1): 83-90. |  |
|  | LI B J, GE W J, SHAN P T, et al. [Pharmacokinetics and tissue distribution of four alkaloids in Ermiao Pills and Sanmiao Pills in normal and arthritic model rats] [J]. Zhongguo Zhong Yao Za Zhi, 2023, 48(7): 1943-50. |  |
|  | LIU J, PAN J, WANG Y, et al. Component analysis of Chinese medicine and advances in fuming-washing therapy for knee osteoarthritis via unsupervised data mining methods [J]. J Tradit Chin Med, 2013, 33(5): 686-91. |  |
|  | SIU W S, SHUM W T, CHENG W, et al. Topical application of Chinese herbal medicine DAEP relieves the osteoarthritic knee pain in rats [J]. Chin Med, 2019, 14: 55. |  |
| Lack of  outcome  indicator | Dai Weiwei,Wang Libo,Li Junhao et al. The active component β-ecdysterone of cowberry interferes with glucocorticoid osteoporosis through Connexin43-AKT signaling pathway[C]// Chinese Society of Integrative Medicine, Orthopaedic and Traumatology Branch. Compilation of papers from the 24th Annual Conference of Chinese and Western Medicine in Orthopaedics and Traumatology. [publisher unknown],2017:441-442. | 8 |
|  | Gao Weihui, Xiang Yanhua, Liu Yun, Deng Guiming, Lin Jie, Lin Wang Senzhi, He Yao. Experimental study on the intervention of Chinese herbal medicine Eucommia-cowberry combination on osteoporosis in de-ovulatory rats[J]. Journal of Hunan University of Traditional Chinese Medicine,2016,36(06):43-46. |  |
|  | Qu Ning-Ning. Study on the material basis and mechanism of action of Zuo Gui Wan against osteoporosis in de-ovalized rats[D]. Liaoning University of Traditional Chinese Medicine,2016. |  |
|  | Jia Yumin. Study on the mechanism of action of Aconite for kidney and bone formula in the treatment of osteoporosis in rats[D]. Hubei University of Traditional Chinese Medicine, 2013. |  |
|  | Zhang Lei. Study on the mechanism of bone paralysis compound intervention on degenerated chondrocytes in the knee joint of C518 rats[D]. Yunnan College of Traditional Chinese Medicine, 2013. |  |
|  | TANG Junfeng,LU Ya'e,HU Xiqin. Effect of Urtica dioica formula on bone density in de-ovulatory rats[J]. Shaanxi Traditional Chinese Medicine,2011,32(02):240-242. |  |
|  | Dong Qunwei,Chen Zhifeng,Sun Fenyong. Bovine knee destructor promotes the proliferation of mesenchymal stem cells in de-ovulatory rats[J]. Guangdong Medicine,2010,31(01):61-63. |  |
|  | Dong Qunwei,Chen Zhifeng,Chen Shaoqing,Sun Fenyong,Hu Lingping,Hong Manjie,Liang Chao. The therapeutic effect of bovine knee deoxysterone on osteoporosis in de-ovulatory rats[J]. Journal of Guangdong Pharmaceutical University,2009,25(05):512-515. |  |
| Double publication | Dai Weiwei, Exploring the effector mechanism of the active ingredient β-ecdysterone in cowberry based on the dynamic changes of glucocorticoid-induced autophagy and apoptosis in mouse osteoblasts. Shanghai, Longhua Hospital, Shanghai University of Traditional Chinese Medicine, 2017-12-12. | 2 |
